# Large-Scale Generation of Human Allospecific Induced Tregs With Functional Stability for Use in Immunotherapy in Transplantation

**DOI:** 10.3389/fimmu.2020.00375

**Published:** 2020-04-02

**Authors:** Evelyn Katy Alvarez-Salazar, Arimelek Cortés-Hernández, Saúl Arteaga-Cruz, Josefina Alberú-Gómez, Gloria Soldevila

**Affiliations:** ^1^Departamento de Inmunología, Instituto de Investigaciones Biomédicas, Universidad Nacional Autónoma de México, Ciudad de México, Mexico; ^2^Tecnológico de Monterrey, Escuela de Medicina y Ciencias de la Salud, Monterrey, Mexico

**Keywords:** induced Tregs, alloimmunity, immunotherapy, tolerance induction, transplantation

## Abstract

Regulatory T cells play an important role in the control of autoimmune diseases and maintenance of tolerance. In the context of transplantation, regulatory T cells (Tregs) have been proposed as new therapeutic tools that may induce allospecific tolerance toward the graft, avoiding the side effects induced by generalized immunosuppressors. Although most clinical trials are based on the use of thymic Tregs in adoptive therapy, some reports suggest the potential use of *in vitro* induced Tregs (iTregs), based on their functional stability under inflammatory conditions, indicating an advantage in a setting of allograft rejection. The aim of this work was to generate and expand large numbers of allospecific Tregs that maintain stable suppressive function in the presence of pro-inflammatory cytokines. Dendritic cells were derived from monocytes isolated from healthy donors and were co-cultured with CTV-labeled naïve T cells from unrelated individuals, in the presence of TGF-β1, IL-2, and retinoic acid. After 7 days of co-culture, proliferating CD4^+^CD25^++^CTV^−^ cells (allospecific iTregs) were sorted and polyclonally expanded for 6 weeks in the presence of TGF-β1, IL-2, and rapamycin. After 6 weeks of polyclonal activation, iTregs were expanded 230,000 times, giving rise to 4,600 million allospecific iTregs. Allospecific iTregs were able to specifically suppress the proliferation of autologous CD4^+^ and CD8^+^ T cells in response to the allo-MoDCs used for iTreg generation, but not to third-party allo-MoDCs. Importantly, 88.5% of the expanded cells were CD4^+^CD25^+^FOXP3^+^, expressed high levels of CCR4 and CXCR3, and maintained their phenotype and suppressive function in the presence of TNF-α and IL-6. Finally, analysis of the methylation status of the FOXP3 TSDR locus demonstrated a 40% demethylation in the purified allospecific iTreg, prior to the polyclonal expansion. Interestingly, the phenotype and suppressive activity of expanded allospecific iTregs were maintained after 6 weeks of expansion, despite an increase in the methylation status of the FOXP3 TSDR. In conclusion, this is the first report that demonstrates a large-scale generation of allospecific iTregs that preserve a stable phenotype and suppressor function in the presence of pro-inflammatory cytokines and pave the way for adoptive cell therapy with iTregs in transplanted patients.

## Introduction

Regulatory T cells (Tregs) are a subtype of CD4^+^ T cells that play an important role in the control of autoimmune diseases and maintenance of immune homeostasis and that are distinguished by the expression of CD25 and the transcription factor FOXP3 (1, 2). Tregs can be generated in the thymus, with the combination of strong antigenic signals and high costimulation, named thymus-derived regulatory T cells [tTregs; frequently known as natural Tregs (nTregs)]. In addition, other subtypes of Tregs can also be generated in the periphery from naïve T cells after encounter with own or foreign antigens, under conditions of limiting costimulation and are known as periphery-derived Tregs (pTregs), when they are generated *in vivo*, or induced Tregs (iTregs), when they are generated *in vitro* (3, 4). Although both subpopulations (thymic and peripheral) develop in different anatomical locations, they express common superficial receptors associated with their functions, such as CTLA-4 (also known as CD152), GITR, CD103, and ICOS (5). Analysis of T cell receptor (TCR) repertoires showed that pTregs and tTregs recognize different antigens, which explains why both subpopulations are necessary in the maintenance of tolerance (6, 7). tTregs express a TCR repertoire with a bias for self, being most important in the prevention of autoimmunity, while pTregs are present to moderate response to foreign antigens like those from microbiota or diet, as well as fetus-derived antigens during pregnancy (8). Therefore, the contributions to the immune regulation by both subpopulations are not redundant, and therefore, the participation of both is required to effectively suppress immune response (9, 10).

Studies in animals and humans highlight the role of Tregs in the induction of tolerance to allografts, which motivated the development of strategies to expand or generate Tregs for therapeutic use (11, 12). Although most clinical trials have focused on the use of Tregs of thymic origin as adoptive therapy, there are studies that support the potential use of *in vitro* iTregs due to their superior functional stability and efficiency in inflammatory conditions. In this context, it has been reported that iTregs but not tTregs are resistant to Th17 cell conversion when stimulated with IL-6 (13) and that transferred TGF-β1-induced iTregs are more stable and functional than tTregs in mice with established autoimmunity (14). In addition, it has been demonstrated that *in vitro* iTregs are superior than tTregs in their ability to migrate to sites of inflammation in a model of peritonitis, as FOXP3^+^-inducible Tregs but not tTregs efficiently interact with endothelial selectins and transmigrate through endothelial monolayers, releasing secretion products that blocked acute inflammation (15). These data suggest an advantage in the use of iTregs to control the alloimmune response, as the transplanted organ creates an inflammatory environment that could result in the rejection of the allograft.

A potential problem for the use of *in vitro* iTregs is the reported instability of their phenotype and methylation status of the non-coding region of *FOXP3* gene (16), as a result of the conditions used for their generation, such as an overstimulation with polyclonal stimulus (17) or short-time cultures in the absence of stabilization agent, such as rapamycin (RAPA) and all-trans retinoic acid (ATRA). Therefore, we hypothesized that by providing the cells with an adequate signaling threshold during activation and employing the reported agents to increase the expression and stability of FOXP3, it would be feasible to achieve adequate numbers of the desired phenotypic and functional stability to be used in the clinical therapy. Thus, the aim of the present study was to develop a novel protocol to generating large numbers of allospecific Tregs from human naïve T cells, with a stable phenotype and suppressor function in the presence of pro-inflammatory cytokines. Our results support the development of a successful protocol for obtaining large numbers of stable iTregs and will pave the way for their use in allospecific Treg-based immunotherapy.

## Materials and Methods

### Sample Collection

Blood samples were provided by the Blood Bank of Instituto Nacional de Enfermedades Respiratorias, Mexico City, after informed consent was signed, in accordance with the protocol approved by the Institutional Research and Ethics Committee. Peripheral blood mononuclear cells (PBMCs) were isolated from buffy coat preparations from healthy donors by standard Ficoll-Paque™ Plus density-gradient centrifugation (Sigma-Aldrich, St. Louis, MO). A fraction of PBMCs were resuspended in FBS 10% dimethyl sulfoxide (DMSO, Sigma-Aldrich) and immediately frozen at −70°C and 24 h later were stored in liquid nitrogen. The second fraction of PBMCs was used in subsequent cell culture assays.

### Reagents

Anti-Human CD25-PE/Cy5 was purchased from BD Biosciences, (San Jose, CA). Anti-Human CD4-PE, anti-Human CD4-APC, anti-Human CD8-PE/Cy7, and FOXP3/Transcription Factor Staining Buffer Set were purchased from Tonbo Biosciences (San Diego, CA). Anti-Human CTLA-4-PE/CY7, anti-Human CD45RA-APC/Fire™750, anti-Human CCR7-PerCP/Cy5.5, anti-Human CXCR3-VB421, anti-Human CCR4-BV711, and Zombie Aqua™ Fixable Viability Kit were purchased from Biolegend (San Diego, CA). Anti-Human FOXP3-Alexa Fluor®647 was purchased from Beckman Coulter (Brea, CA). Carboxy fluorescein succinimidyl ester (CFSE) and CellTrace Violet (CTV) were purchased from Thermo Fisher Scientific (Waltham, MA). Recombinant Human GM-CSF, IL-2, IL-4, IL-6, TGFβ-1, and TNF-α cytokines were from PeproTech (New Jersey, USA). Some assays were performed in RPMI 1640 (Thermo Fisher Scientific) medium supplemented with 20% fetal bovine serum (FBS, Thermo Fisher Scientific), 2 mM L-glutamine (Thermo Fisher Scientific), 10 mM HEPES, and 10 mM antibiotic/antimycotic (Thermo Fisher Scientific). All cell culture assays were performed at 37°C and 5% CO_2_ conditions.

### Generation of Allospecific iTregs With Allogeneic Monocyte-Derived Dendritic Cells

CD14^+^ monocytes were isolated from PBMCs by positive selection using magnetic MicroBeads (Miltenyi Biotec, Bergisch Gladbach, NRW) according to the manufacturer's protocol and washed twice with RPMI medium. The CD14^+^ cells (1–2 × 10^6^) were cultured for 8 days in RPMI medium supplemented with 10% human AB serum, 50 ng/ml of GM-CSF, and 50 ng/ml of IL-4 into 48-well plates. Monocyte-derived dendritic cells (Mo-DCs) were irradiated with 3,000 rads prior to the functional assays.

For naïve T cell isolation, PBMCs from other healthy individual (allogeneic) were stained with anti-CD4, anti-CD25, and anti-CD45RA monoclonal for 20 min at 4°C in the dark, washed twice with phosphate-buffered saline (PBS) and resuspended in PBS. A CD4^+^CD25^−^CD45RA^+^ gate was used for sorting naïve T cells using the FACS Aria I sorter (BD Biosciences). The sorted cells were collected in culture medium RPMI 20% FBS, washed twice with PBS 1×, stained with CTV, and resuspended in OpTmizer™ CTS™ T-Cell Expansion culture medium (Thermo Fisher Scientific). Subsequently, CTV-labeled naïve T cells were co-cultured for 7 days with irradiated allogeneic Mo-DCs (naïve T cells: Mo-DC ratio of 10:1) in culture medium supplemented with 5 ng/ml TGF-β1, 100 U/ml IL-2, and 10 nM ATRA (Sigma-Aldrich) into 96-well U bottom plates.

### Polyclonal Expansion of Allospecific iTregs

On day 7 of co-culture of naïve T cells and Mo-DCs, cells were stained with anti-CD25, anti-CD4, and Zombie Aqua™ for 20 min at room temperature in the dark and then washed with PBS 1×. Proliferating T cells (CD4^+^CD25^++^CTV^−^) and non-proliferating T cells (CD4^+^CD25^+^CTV^+^) were sorted by FACS. The sorted cells were collected in RPMI medium 20% FBS, washed twice, and resuspended in OpTmizer™ CTS™ T-Cell Expansion medium supplemented with 50 U/ml IL-2 for 3 days. Then, the cells were cultured with (bead: cells of 1:1–1:2) anti-CD3/CD28-coated beads (Dynabeads Human T-activator CD3/CD28, Thermo Fisher), 100–200 U/ml IL-2, and 100 ng/ml RAPA for 4 days (expansion). On day 4 of expansion, anti-CD3/anti-CD28-coated beads were removed, and cells were washed once and cultured with 50 U/ml IL-2 for 3 days (resting). This scheme (4 days of expansion and 3 days of resting) was repeated for five consecutive weeks. After 6 weeks of expansion, a fraction of allospecific Tregs was cultured without TGF-β1 and/or RAPA (Sigma-Aldrich) for one more week. The other stimuli (anti-CD3/anti-CD28-coated beads and IL-2) were maintained following the expansion and resting scheme described above.

### Analysis of iTreg Stability in the Presence of Pro-Inflammatory Cytokines

After 4 weeks of expansion, a fraction of allospecific Tregs were cultured for 2 weeks in the presence of 10 ng/ml of recombinant human IL-6 and TNF-α cytokines (PeproTech) using the same scheme of expansion and resting. Then, cells were surface and intracellular stained, and suppression assays were performed.

### Surface and Intracellular Staining

Allospecific iTregs were stained with anti-CD25, anti-CD4, anti-CCR7, anti-CCR4, anti-CXCR3, and Zombie Aqua™ for 20 min at room temperature in the dark and washed with PBS 1×. For intracellular staining, cells were permeabilized with Fixation/Permeabilization solution at room temperature for 1 h and washed once with Permeabilization buffer 1×. Cells were incubated with anti-FOXP3 and anti-CTLA-4 for 30 min at 4°C in the dark and washed twice with Permeabilization buffer 1×. Samples were acquired with on Attune® Acoustic Focusing Cytometer (Thermo Fisher Scientific), and data were analyzed with FlowJo vX.0.7 software (Tree Star, Covington, KE, USA).

### Allospecific Suppression Assay

At weeks 4 and 6 of allospecific iTreg expansion, we evaluated their suppressor capacity by analyzing the inhibition of T cell proliferation. Eight days prior to the suppression assays, allogeneic or third-party Mo-DCs were generated as described above. Autologous CD3^+^ T cells (responder T cells, Tresp) were isolated using Pan T cell Isolation Kit (Miltenyi Biotec) following the manufacturer's protocol and labeled with CFSE. Then, CD3^+^ T cells were co-cultured with allospecific or non-allospecific iTregs (labeled with CTV) at several Tregs:Tresp ratios (0:1, 1:2, 1:4, 1:8, and 1:16) and stimulated with allogeneic or third-party Mo-DCs at a ratio of 1:4 (Mo-DCs:CD3^+^ T cells). For some assays, co-cultures were stimulated in the presence or absence of 10 ng/ml of IL-6 or TNF-α. After 4 days of co-culture, cells were stained with anti-CD4/anti-CD8 for 20 min at 4°C in the dark and washed twice. Cells were acquired with on Attune NxT Cytometer (Thermo Fisher Scientific), and data were analyzed with FlowJo vX.0.7 software (Tree Star). The percentage of proliferation on gated CD4^+^ or CD8^+^ T cells was determined by CFSE dilution after exclusion of CTV-labeled Tregs. The percentage of suppression was calculated using the following formula:

[(Proliferation of Tresp without Tregs – Proliferation of Tresp with Tregs)/Proliferation of Tresp without Tregs] × 100.

### Cytokine Production Assay

The level of cytokines IL-2, IL-10, and IFN-γ in the culture supernatants from the suppression assays was measured using the kit LEGENDplex (Biolegend), according to the manufacturer's guidelines. The samples were acquired on the flow cytometer Attune® NxT. Cytokine concentrations were determined using the standard curve generated in the same assay.

### FOXP3 TSDR DNA Methylation Analysis

Methylation of Treg-specific demethylation region (TSDR) of FOXP3 gene was evaluated in expanded allospecific Treg at day 0, and at the 4 and 6th week of expansion. As control, CD4^+^CD25^−^CD45RA^+^ T cells were included. Sodium bisulfite modification of genomic DNA was carried out using the EZ DNA Methylation direct Kit (Zymo Research Corp, Irvine, CA) according to the manufacturer's protocol. Bisulfite-treated DNA was PCR amplified using the following primers: p-5′-TGATTTGTTTGGGGGTAGAGGATTTAGAG-3′ and o-5′-TATCACCCCACCTAAACCAAACCTACTACA-3′. Amplified DNA product was gel purified using the QIAEX II gel extraction kit (Qiagen, Hilden, NRW) and cloned into pGEM-T easy vector (Promega, Madison, WI). Competent bacterial cells were transformed with recombinant vector and positive colonies were selected, from which recombinant plasmid DNA was purified and sequenced with 3500 Genetic Analyzer (Thermo Fisher Scientific). Sequences were analyzed using the Bioedit Software 7.2.5 (Ibis Biosciences, Carlsbad, CA).

### Statistical Analysis

The statistical analysis was performed using Prism 6.0 software (GraphPad Software, San Diego, CA). The Kolmogorov–Smirnov test was used to evaluate the distribution of each group. Paired and unpaired Student *t*-tests were used for comparing normally distributed data; Wilcoxon matched-pairs signed rank test or Mann–Whitney tests were used for non-normally distributed data. Differences between more than two groups were calculated using the one-way ANOVA test for normally distributed data and Kruskal–Wallis test for non-normally distributed data. Graphs are expressed as mean ± standard error of the mean (SEM). *P* < 0.05 was considered significant.

## Results

### Immature Mo-DCs Induce the Generation of Allospecific Tregs

**Figure 1:**
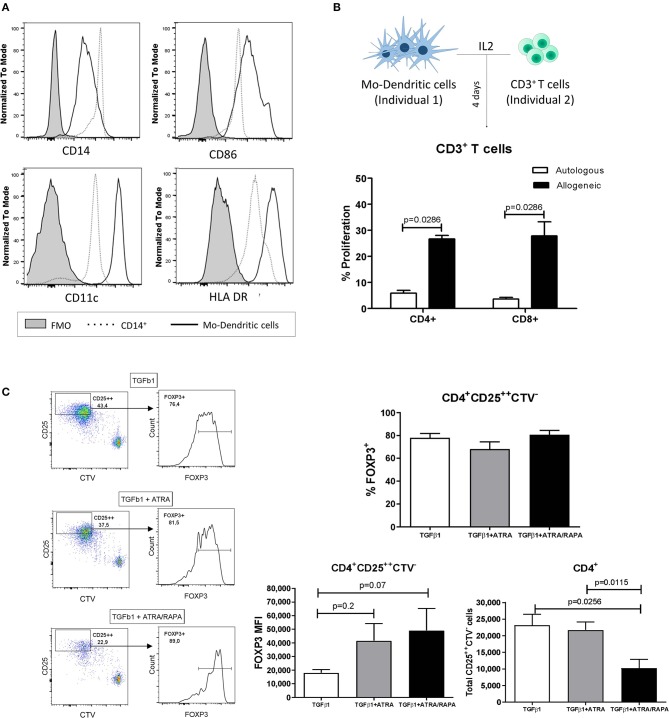
Monocyte-derived dendritic cells generate *de novo* allospecific induced Tregs. Dendritic cells were derived from peripheral CD14^+^ monocytes with GM-CSF and IL-4 for 8 days. **(A)** Expression of representative markers on Mo-DCs was determined by flow cytometry. Mo-DCs express higher levels of CD86, CD11c, and HLA-DR compared to CD14^+^ monocytes. **(B)** Mo-DCs were able to induce CD4^+^ and CD8^+^ T cell proliferation in co-culture with T cells from unrelated individuals. **(C)** Mo-DCs were co-cultured with labeled CTV naïve T cells in the presence of TGF-β1 and all-trans retinoic acid (ATRA) and/or rapamycin (RAPA). The great majority of proliferating CD4^+^CD25^++^CTV^−^ cells from the co-culture between allogeneic Mo-DCs and naïve T cells express the transcription marker FOXP3 (left panels) in all conditions. Although the frequency of iTregs (identified as CD25^++^FOXP3^+^) is similar among the three conditions evaluated (bar graph, upper right), the expression of FOXP3 [mean fluorescence intensity (MFI)] is slightly higher in the conditions with ATRA and/or RAPA. A greater number of cells are obtained in the co-cultures with TGF-β1 + ATRA, from which CD4^+^CD25^++^CTV^−^ cells can be sorted with greater efficiency to go on the subsequent assays (bar graphs, lower right). MFI values were calculated by subtracting the FMO (fluorescence minus one) control of the indicated antibody from the MFI value of each individual. Data represent the mean ± SEM from *n* = 5 donors from two independent experiments. The statistical analysis was performed using the non-parametric Mann–Whitney test, two-tailed. *P* < 0.05 was considered statistically significant.

To generate allospecific iTregs, dendritic cells were derived from peripheral blood CD14^+^ monocytes purified from healthy donors after culture in the presence of GM-CSF and IL-4 for 8 days. Non-adherent cells were stained with anti-HLA-DR, anti-CD86, and anti-CD11c, and analyzed by flow cytometry, showing the expression of characteristic DC surface markers (Figure 1A). Mo-DCs were then co-cultured with CFSE-labeled allogeneic CD3^+^ T cells, and CD4^+^ and CD8^+^ T cell proliferative responses were measured (Figure 1B). Next, to establish the optimal methodology to generate iTregs, we co-cultured CTV-labeled naïve T cells with allogeneic Mo-DCs in three different conditions: TGF-β1 (1), TGF-β1 + ATRA (2), or TGF-β1 + ATRA/RAPA (3) (Figure 1C, dot plots and histograms, left). iTregs (proportion of CD4^+^CD25^+^FOXP3^+^ cells) were generated in all these conditions, although the highest expression of FOXP3 [mean fluorescence intensity (MFI)] was observed in the presence of ATRA [conditions (2) and (3)], showing no differences between them (Figure 1C, bar graphs, right). However, greater cell numbers were observed in condition (2) compared to condition (3), possibly due to the effect of RAPA, an inhibitor of cell proliferation (Figure 1C, bar graphs, right). Therefore, this condition (2) was chosen for the generation of allospecific iTregs.

### Polyclonally Expanded iTregs Express High Levels of FOXP3 and CD25

**Figure 2:**
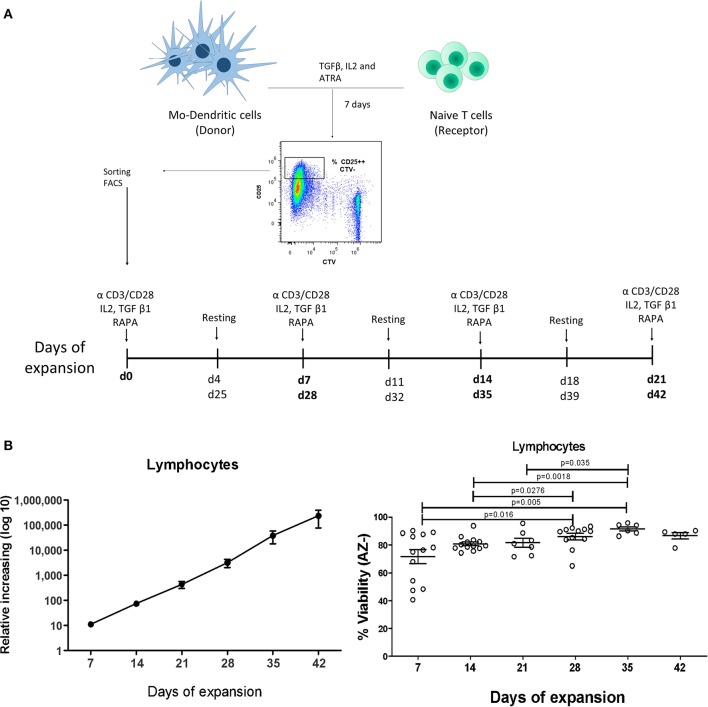
Purification and expansion of allospecific iTregs. **(A)** Schematic representation of the protocol used for the generation and expansion of allospecific iTregs. Mo-DCs were co-cultured with CTV-labeled naïve T cells from an unrelated individual for 7 days and proliferating CD4^+^CD25^++^CTV^−^ cells (allospecific iTregs) were sorted by FACS and polyclonally expanded for 6 weeks. The phenotype, suppressor function, and cytokine production were evaluated. **(B)** Cell counts (left graph) and viability (right graph) of Tregs expanded for 6 weeks. Data represent the mean ± SEM from *n* = 5–15 donors from five independent experiments. The statistical analysis was performed using the non-parametric Mann–Whitney test, two-tailed. *P* < 0.05 was considered statistically significant.

**Figure 3:**
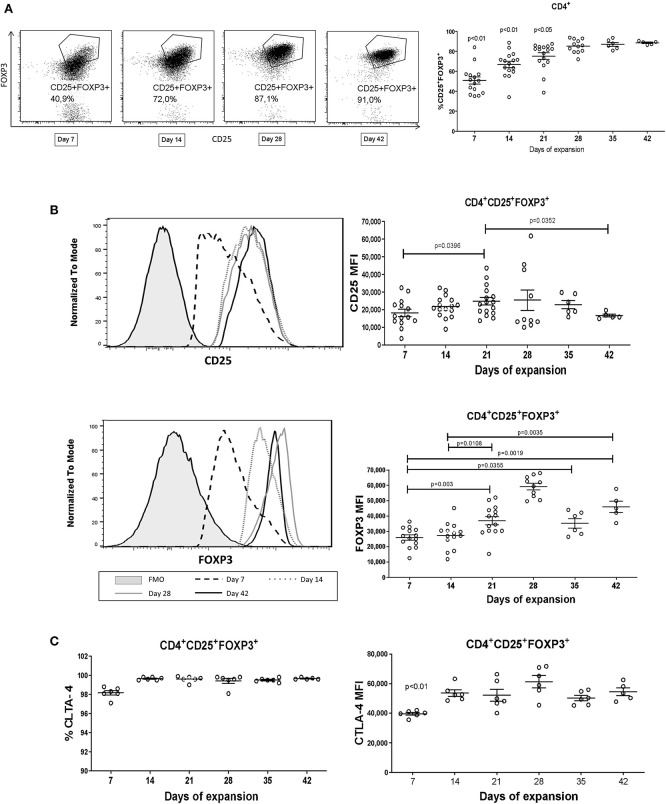
Long-term expansion of iTregs increases the proportion of CD25^+^FOXP3^+^ and expression of FOXP3. **(A)** Representative dot plots of allospecific CD25^+^ FOXP3^+^ iTregs expanded for 7, 14, 28, and 42 days. The percentage of CD4^+^CD25^+^FOXP3^+^ iTregs increased during the expansion culture reaching 90% at 42 days. **(B)** Increase in the expression of CD25 and FOXP3 (left graphs) in expanded iTregs for 6 weeks. **(C)** The expanded iTregs were positive for CTLA-4 with intensity increases after the 2nd week of expansion. MFI values were calculated as above. Data represent the mean ± SEM from *n* = 5–14 donors from five independent experiments. The statistical analysis was performed using the non-parametric Mann–Whitney test, two-tailed. *P* < 0.05 was considered statistically significant.

**Figure 4:**
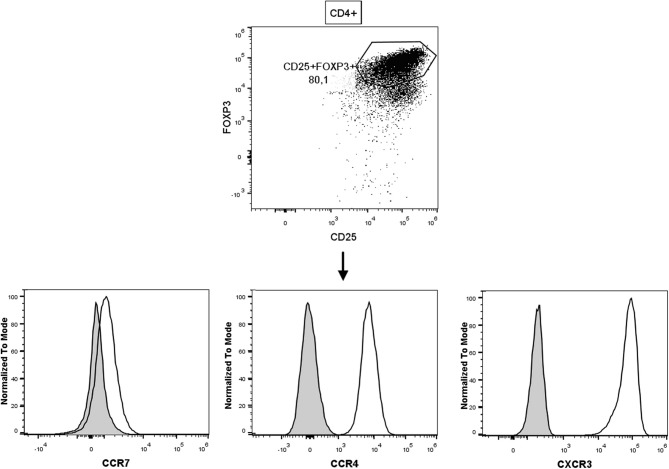
Expanded allospecific iTregs express chemokine receptors related to migration to inflamed tissue. Representative dot plot of allospecific CD25^+^FOXP3^+^ iTregs expanded for 28 days. Histograms show the expression level of each chemokine receptor (CCR7, CCR4, and CXCR3) from allospecific iTregs (black solid line); filled histograms denote a fluorescence minus one control (FMO). Data are representative of at least five different healthy donors.

Proliferating CD4^+^CD25^++^CTV^−^ cells (allospecific iTregs) from the co-cultures containing TGF-β1 + ATRA were sorted and expanded with CD3/CD28-coated beads and IL-2 for 6 weeks (Figure 2A). On the sixth week, these cells had expanded 230,000-fold expansion times, giving rise to 4,600 million iTregs from 20,000 initial sorted proliferating CD4^+^CD25^++^CTV^−^ cells (Figure 2B, left panel) and reaching a viability close to 95% (Figure 2B, right). At that time, 88.5% of the expanded cells were CD4^+^CD25^+^FOXP3^+^ (Figure 3A) with an increased FOXP3 expression up to the fourth week of expansion (Figure 3B, bottom panel). Expression of CD25 (Figure 3B, upper panel) and CTLA-4 (Figure 3C, right panel) was increased from the second week of culture, after which no noticeable differences were observed in the following weeks. Finally, all cells were positive for CTLA-4 (Figure 3C, left panel). Interestingly, expanded allo-iTregs expressed high levels of chemokine receptors CCR4 and CXCR3 and low levels of CCR7, which is highly relevant for their potential functionality *in vivo* (Figure 4).

### Expansion in the Presence of Pro-Inflammatory Cytokines Does Not Alter the Frequency and Expression of FOXP3 and CD25

**Figure 5:**
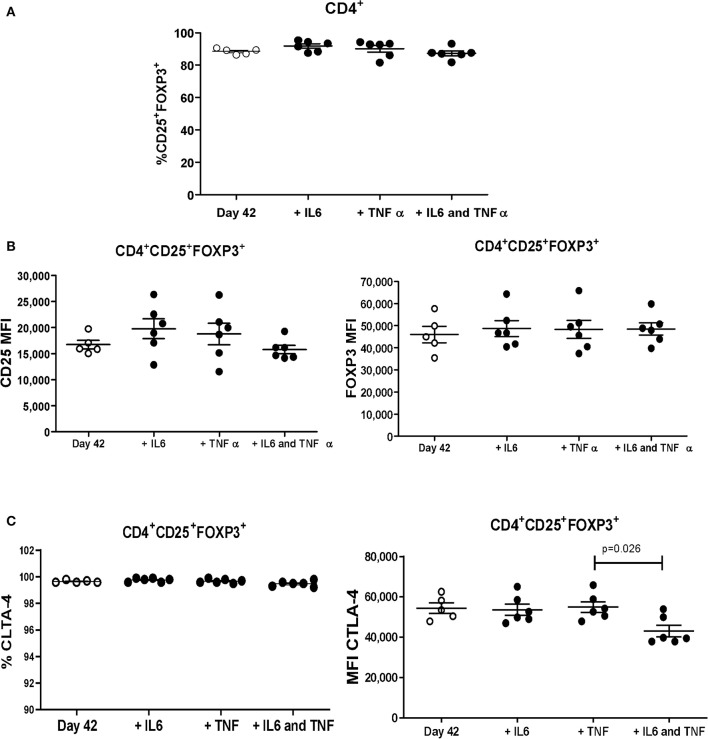
Allospecific iTregs expanded in the presence of pro-inflammatory cytokines maintain the frequency and levels of expression of CD25 and FOXP3. Allospecific CD4^+^CD25^++^CTV^−^ cells (Figure 2) expanded for 4 weeks were polyclonally stimulated for two additional weeks with IL-6 and TNFα. **(A,B)** No differences in frequency of CD25^+^FOXP3^+^ were observed when the iTregs were expanded in the presence of IL-6 and/or TNF-α **(A)**; similar results were observed in the expression of CD25 and FOXP3 **(B)**. **(C)** Percentage of CTLA-4^+^ cells within the CD25^+^FOXP3^+^ population is maintained in the presence of cytokines, although the expression levels were significantly diminished. MFI values were calculated as above. Data represent the mean ± SEM from *n* = 5–6 donors from three independent experiments. The statistical analysis was performed using the non-parametric Mann–Whitney test, two-tailed. *P* < 0.05 was considered statistically significant.

After 4 weeks of expansion, iTregs were cultured with pro-inflammatory cytokines IL-6 and/or TNF-α for two additional weeks. The frequency of CD25^+^ FOXP3^+^ population and the expression of FOXP3, CD25, and CTLA-4 was maintained in the presence of the indicated cytokines, when compared with control iTregs (without cytokines) (Figure 5), suggesting that these cells may be stable in an inflammatory environment.

### iTregs Expanded for 6 Weeks Exert Allospecific Suppression, Which Is Not Affected by Pro-Inflammatory Cytokines

**Figure 6:**
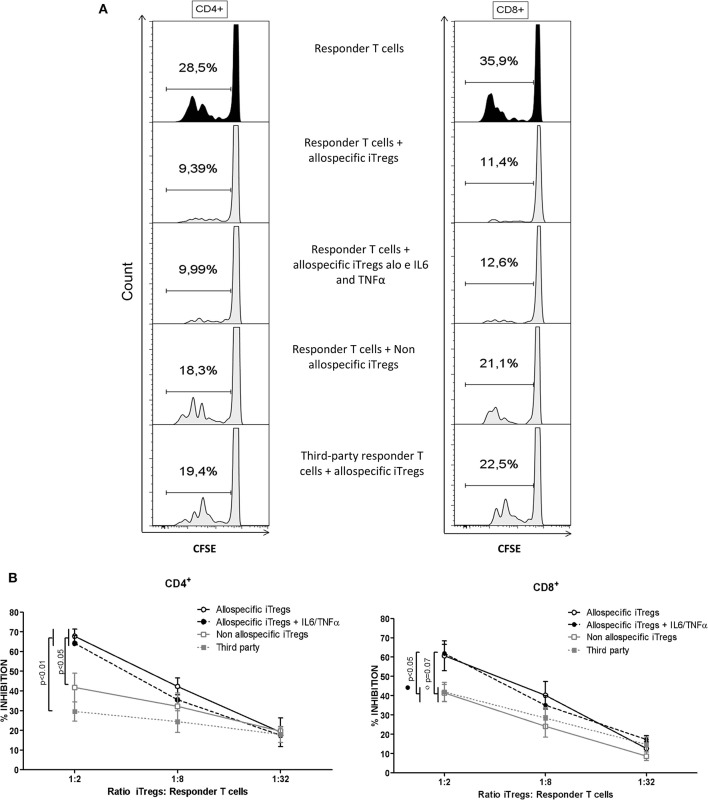
Allospecific suppression mediated by long-term expanded iTregs is not affected by pro-inflammatory cytokines. Proliferating CD4^+^CD25^++^CTV^−^ cells (Figure 2) expanded for 4 weeks were stimulated for two additional weeks with IL-6/TNFα and were added to co-cultures of T cells/mo-DCs. **(A)** Histograms show the proliferation of CD3^+^ T cells in the absence (responder T cells) or the presence of the allospecific expanded iTregs without the addition of cytokines during polyclonal expansion (responder T cells + allospecific iTregs) or with the addition of the cytokines IL-6 + TNF-α during the next 2 weeks of expansion (responder T cells + allospecific iTregs and IL-6/TNF-α). As controls, allospecific iTregs and stimulating DCs from unrelated individuals (third-party responder T cells) were considered. The numbers indicate the percentage of proliferation, when iTregs:responder T cells are put into the proportion of 1:2. **(B)** Graph showing the percentage of inhibition of the proliferation of responder T cells (CD3^+^CD4^+^ and CD3^+^CD8^+^) against the different conditions mentioned and into different proportions of iTregs:T responders. Suppression was calculated as relative inhibition using the following formula: [(Tresp proliferation without Tregs – Tresp proliferation with Tregs)/Tresp proliferation without Tregs] × 100. Each dot indicates the mean ± SEM from *n* = 5 donor from three independent experiments of each condition tested. Statistical analysis was performed using non-parametric Mann–Whitney test, two-tailed. *P* < 0.05 was considered statistically significant.

To evaluate whether expanded iTregs are capable of inducing allospecific suppression, allogeneic Mo-DCs were co-cultured with responder CD3^+^ T cells and allospecific iTregs. Interestingly, allospecific iTregs were able to significantly suppress the proliferation of autologous CD4^+^ and CD8^+^ T cells at a ratio of 1:2 (Treg: Tresp), in response to the allo-MoDCs used for iTreg generation (Figure 6). Also, these iTregs did not suppress the proliferation of CD3^+^ T cells that responded to other antigens (third-party allo-MoDCs), indicating that expanded iTregs are only specific for the donor alloantigens. Finally, the inhibition of T cell proliferation exerted by iTregs was not affected when pro-inflammatory cytokines IL-6/TNF-α were added during their last 2 weeks of expansion (Figure 6) or when the cytokines were added during the suppression assay (Supplementary Figure S1), suggesting that these expanded iTregs are functionally stable in inflammatory conditions.

### Long-Term Expansion of iTregs Modulates Production of IL-2, IL-10, and IFN-γ to Suppress the Proliferation of Responder T Cells

**Figure 7:**
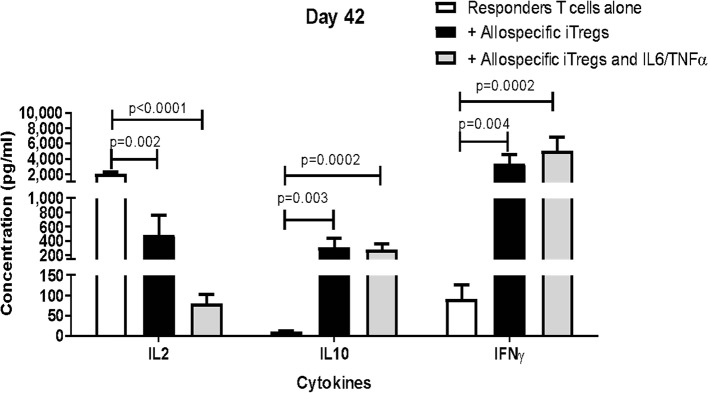
Allospecific expanded iTregs induce production of IL-10 and IFN-γ and downregulate IL-2 to suppress the proliferation of responder T cells. The concentrations of cytokines were measured in the supernatants of the suppression assays. The graphs show the production of cytokines from responder T cells alone (white bar), co-cultures of responder T cells with allospecific iTregs expanded for 6 weeks (black bar), and co-cultures with iTregs expanded for the last 2 weeks with IL-6 and TNF-α (gray bar, lower panel). The measurement of cytokines in the co-cultures indicates a reduction in the production of IL-2 and an increase in the production of IL-10 and IFN-γ in the presence of iTregs, compared to responder T cells alone. Data represent mean ± SEM from *n* = 5 donors from three independent experiments. The statistical analysis was performed using the non-parametric Mann–Whitney test, two-tailed. *P* < 0.05 was considered statistically significant.

We analyzed cytokine production in the culture supernatants of the same suppression assays of iTregs expanded for 4 (Supplementary Figure S2) or 6 (Figure 7) weeks. We found a significant decrease in the production of IL-2 and increased levels of IL-10 and IFN-γ, compared to the levels found in responders T cells alone, suggesting that IL-2 consumption and production of immunosuppressive cytokines may be involved in the suppressive mechanisms used by allospecific iTregs. This cytokine production was not affected when iTregs were expanded with pro-inflammatory cytokines IL-6/TNF-α during their last 2 weeks of expansion (Figure 7, gray bar), in agreement with the functional results showing that expanded iTregs can exert their suppressive function in an inflammatory setting.

### Rapamycin Is Required to Maintain the Phenotype of Expanded Allospecific iTregs

**Figure 8:**
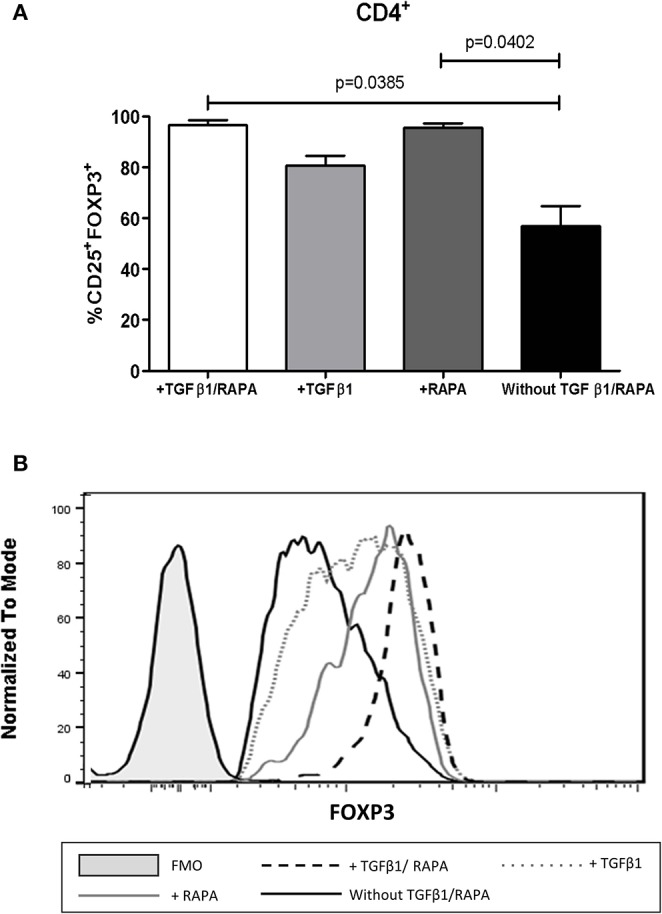
Rapamycin is required to maintain the phenotype and expression of FOXP3 on expanded allospecific iTregs. Proliferating CD4^+^CD25^++^CTV^−^ cells (Figure 2) expanded for 6 weeks were stimulated for an additional week with four conditions: (1) with TGF-β and rapamycin (TGF-β + RAPA); (2) only TGF-β (+ TGF-β); (3) only RAPA (+ RAPA); (4) absence of both TGF-β and RAPA (without TGF-β/RAPA). iTreg cells showed an altered proportion of CD25^+^FOXP3^+^ **(A)** and FOXP3 expression **(B)** when TGF-β1 and/or RAPA was removed from expansion cultures, and the presence of TGF-β1 alone was not sufficient to maintain the regulatory phenotype. In contrast, RAPA was sufficient to maintain the percentage of CD25^+^FOXP3^+^ Tregs and FOXP3 expression in the expanded iTregs. Data represent the mean ± SEM from *n* = 5 donors from three independent experiments. The statistical analysis was performed using the non-parametric Mann–Whitney test, two-tailed. *P* < 0.05 was considered statistically significant.

To evaluate the contributions of TGF-β1 and RAPA in maintaining iTreg phenotype, these cells were cultured in the absence of these stimuli. When TGF-β1 and/or RAPA was removed from expansion cultures, iTregs showed an altered phenotype and FOXP3 expression, since the presence of TGF-β1 alone was not sufficient to maintain the regulatory phenotype (Figure 8). In contrast, RAPA was sufficient to maintain the percentage of CD25^+^FOXP3^+^ Tregs and FOXP3 expression in the expanded iTregs (Figure 8), suggesting the importance of RAPA in maintaining the stability and function of these iTregs for a future adoptive therapy.

### iTregs Display Partial Demethylation of TSDR FOXP3 Gene

**Figure 9:**
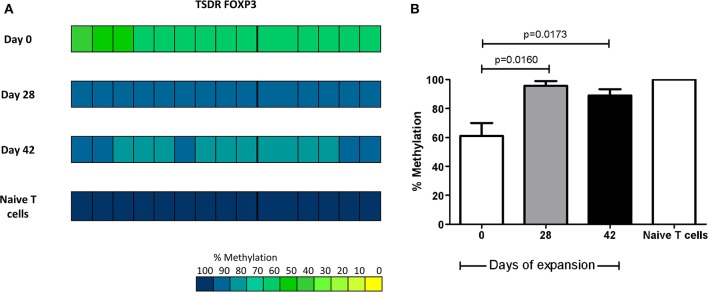
iTregs display partial demethylation of TSDR FOXP3 gene. CpG methylation analysis of TSDR region in FOXP3 gene was evaluated from proliferating CD25^hi^ cells before (Day 0) and after 4 or 6 weeks of expansion (five individuals per group/six clones per individual. **(A)** The panel shows the average of methylation status within TSDR FOXP3 from the three evaluated groups. Naïve T cells display a completely methylated TSDR and were used as negative control. Each square represents one CpG analyzed. Methylation color code ranges from yellow (0% methylation) to blue (100% methylation) according to the color scale (lower right). **(B)** Recently converted iTregs isolated on day 7 of the co-culture with Mo-DCs (Day 0 of the polyclonal expansion) display a partial demethylation of TSDR region (≈40%); however, when the cells were polyclonally expanded for 4 and 6 weeks, the demethylation is reduced up to ≈5 and ≈10%, respectively. Percentage of methylation from each evaluated group, represented as the ratio between the numbers of demethylated cytosines and the total number of sequenced CpG sites within the TSDR region. Data are presented as mean of the percentage of methylation ± SEM of five individuals (*n* = 5) per group (six clones per individual). The statistical analysis was performed using the non-parametric Mann–Whitney test, two-tailed. *P* < 0.05 was considered statistically significant.

To evaluate whether the observed stability in expanded iTregs was associated to their demethylation status of TSDR FOXP3, we proceeded to sequence individual clones obtained from iTregs, after amplification and cloning of PCR products amplified from bisulfite-converted DNA samples. Analysis of the 15 CpGs sites included within the TSDR region was performed to determine the methylation status from each individual clone, which was averaged and are shown in color code ranging from yellow (0% methylation) to blue (100% methylation) (Figure 9A). As shown, recently converted iTregs isolated on day 7 of the co-culture with Mo-DCs (Day 0 of the polyclonal expansion) display a partial demethylation of TSDR region (≈40%); however, when the cells were polyclonally expanded for 4 and 6 weeks, the demethylation is reduced up to ≈5 and ≈10%, respectively (Figure 9B). These data are suggestive of other molecular events contributing to the persistent expression of FOXP3 and stability of expanded iTregs described above, independently of their CpG methylation status.

## Discussion

Tregs not only participate in the maintenance of tolerance to self-antigens and the regulation of the immune response against foreign antigens but also limit alloreactive responses. Therefore, it is plausible to develop different strategies to allow their *de novo* generation and/or *in vitro* expansion, aiming to obtain sufficient numbers of Tregs to control alloreactive T cells present in the receptor, promoting donor-specific tolerance and reducing or avoiding the adverse effects of immunosuppressive drugs.

In this work, we generated allospecific Tregs from naïve T cells from healthy individuals and established a methodology that allowed the generation of a large number of allospecific iTregs with stable phenotype and suppressor function. The methodology was optimized in order to improve the efficiency of induction of Tregs, using a sub-optimal activation of the TCR, by using allogeneic immature dendritic cells and the use of ATRA and RAPA, components reported to promote stability of FOXP3 expression and suppressive function (18, 19). Allospecific responses were obtained by co-culturing naïve CD4^+^ T cells and Mo-DCs from non-related individuals, which, in a transplant scenario, would correspond to the HLA non-identical recipient and donor, respectively. The ability of DCs to process and present multiple epitopes makes them ideal candidates for the generation of specific Tregs for the antigen (20). DCs are scarce in peripheral blood and difficult to maintain in culture, but they can be differentiated *in vitro* from monocytes showing the characteristic features of antigen-presenting cells, including expression of MHC class II and costimulatory molecules. These immature DCs can become mature after stimulation LPS, TNF-α, IFN-γ, or CD40L (21).

Several reports indicate that the presentation of antigens to T cells by immature DCs results in T cell anergy or Treg induction (22). In addition, there is evidence of previous studies describing iTreg generation (23, 24); however, this is the first report in which a large amount of iTregs are obtained with specificity for the antigen of interest. To achieve this, a two-step strategy was used: first, the generation of allo iTregs in co-cultures between naïve T cells and MoDC, and second, isolation of the generated iTregs and their polyclonal expansion for 6 weeks (Figure 2A). In this way, the disadvantages of repetitive restimulation in the same co-culture and the persistence of Tregs with other specificities that may hinder the expansion of allospecific Tregs of interest were avoided.

The isolated cells were expanded with anti-CD3/anti-CD28-coated beads, whose concentrations were standardized to achieve optimal proliferation of iTregs for several weeks without leading to cell death. On the 6th week of expansion, a viability of 90% was reached, resulting in an expansion of allospecific Tregs that reached 230,000 times the initial number, reaching a final number of 4,600 × 10^6^ allospecific iTregs, which is the highest number reported up to now in expansion and/or generation of allospecific Tregs (Figure 2B). According to clinical trials that have employed expanded thymic Tregs with anti-CD3/anti-CD28-coated beads, the average number of cells needed to obtain an adequate suppression is 10–20 × 10^6^ cells/kg per patient (25). Therefore, for a patient of 70 kg, ~700–1,400 × 10^6^ of Tregs would be needed, which could be achieved with the number reached here, and moreover, it would be possible to infuse several doses of these cells. Finally, it was reported that the number required to perform an efficient suppression is lower with allospecific Tregs than with the polyclonal ones (26), making its application as adoptive therapy more feasible.

At the 6th week of expansion, 88.5% of the cells were CD4^+^CD25^+^FOXP3^+^ with an increasing expression of FOXP3 until the 4th week of expansion. It was reported that an effective generation of Tregs requires the inhibition of both complexes that conform the mTOR kinase (mTORC1 and mTORC2), which is only achieved with a prolonged treatment with RAPA (27, 28). This could explain the increasing frequency of CD4^+^CD25^+^FOXP3^+^ T cells over the course of the culture (Figure 3). Also, the fact that iTregs proliferate greatly in the presence of RAPA, despite the fact that this mediator has been reported to inhibit cell proliferation, suggests that these expanded cells acquired the characteristics of Tregs. Indeed, Tregs depend on the IL-2R-mediated JAK/STAT pathway for their proliferation, which is not inhibited by RAPA. Moreover, Tregs express high levels of PTEN, a negative inhibitor of the mTOR pathway, which needs to be downregulated for FOXP3 expression (29, 30). In contrast, conventional T cells require the activation of the PI3K pathway downstream TCR and IL-2 signaling, which is sensitive to the effects of RAPA (31). Our cultures showed that a small subpopulation (around 1–1.5% of cells) were CD25 intermediate/FOXP3 negative, which may represent activated T cells. However, the use of rapamycin ensures the preferential proliferation of Tregs over conventional T cells. It is worth mentioning that expanded iTregs were rested for 3 days with IL-2 alone during each expansion cycle; this strategy was performed to avoid excessive activation during each cycle of proliferation, which could lead to activation-induced cell death, as well as to reduce the overexpression of markers such as CD25 and FOXP3, which could overmask the real frequency and function of iTregs achieved by our protocol.

One of the considerations to take into account for Treg therapy is the fact that in an organ transplant scenario, Tregs must be able to maintain their phenotype and function in an inflammatory cytokine microenvironment. In this context, it has been reported that TNF-α synergizes with IL-6 to prevent transplant tolerance in skin grafting, in addition to increasing the proliferation of effector T cells, making them less susceptible to immune regulation (32) Moreover, IL-6 inhibits Treg differentiation induced by TGF-β1 (33) and TNF-α is capable of affecting FOXP3 expression (34). For this reason, iTregs were expanded for two additional weeks with pro-inflammatory cytokines IL-6 and TNF-α, important in a rejection process (32). The fact that expression of CD25, FOXP3, and CTLA-4 was not affected under these conditions suggested that expanded allospecific iTregs were phenotypically and functionally stable. In an experimental model of autoimmune encephalomyelitis (EAE), it has been reported that iTregs are sensitive to the effect of TNF-α through the inhibition of TGF-β1-induced phosphorylation of SMAD3, reducing its binding to the promoter region of FOXP3 gene (35). Also, the inhibition of PI3K/AKT/mTOR pathway by RAPA (36) may have contributed to the stability observed in the allospecific iTregs generated. On the other hand, it has been reported that treatment of iTregs *in vitro* with IL-6 does not affect the expression of FOXP3 or their suppressor activity compared to thymic Tregs. This was explained by the low expression of IL-6 receptor, which is downregulated by both IL-2 and TGF-β1, suggesting that iTregs are more effective in an inflammatory environment (13). Although IL-6 and TNF-α are considered the most relevant cytokines that iTregs may encounter in the inflammatory milieu of the allograft, we cannot rule out the presence of other cytokines playing a role in a transplantation setting and therefore they may also be considered to further confirm the stability of our allo-iTregs.

Our expanded iTregs were able to suppress the proliferation of autologous CD4^+^ and CD8^+^ T cells in an alloantigen-specific manner (Figure 6). Although the percentage of suppression achieved by our iTregs appears to be lower than that reported by Tu et al. (37), it is worth mentioning that, in this study, the Tregs did not have a resting period prior to the suppression assay and the authors performed the suppression assays based on ^3^H thymidine incorporation, which may lead to overestimated results, as suggested by McMurchy et al. (38).

On the other hand, the suppressive activity of our allo-iTregs correlated with IL-2 consumption and production of IL-10 and IFN-γ (Figure 6). Although IFN-γ is considered a pro-inflammatory cytokine, several reports demonstrate its immunoregulatory role. For example, in an experimental model of GVHD, it was shown that more than 50% of transferred Tregs produce IFN-γ after transplantation and that these Tregs are necessary for the effective prevention of GvHD (39). In addition, using an EAE model, IFN-γ was critical for the conversion of CD4^+^CD25^−^ T cells into Tregs, as *in vitro* treatment of human and murine naïve T cells with IFN-γ led to increased FOXP3 expression and regulatory functions (40).

In addition, it was reported that IFN-γ induces the transcription factor T-bet that promotes the expression of the chemokine receptor CXCR3 on Tregs, which is crucial to counteract Th1-type inflammation (41). Importantly, our data show that the expanded iTregs express high levels of CCR4 and CXCR3, suggesting that they could preferentially migrate into the allograft to suppress the local inflammatory response (42, 43) (Figure 4). Moreover, the expanded allo-iTregs also showed low levels of CCR7, which has been shown to participate in Treg-mediated immune suppression through regulating Treg homing to the graft and positioning to specific niches in the draining lymph nodes (42, 44).

Although our data showed that expanded allospecific iTregs maintained their phenotype and function in the presence of pro-inflammatory cytokines, they required the presence of TGF-β1 and especially RAPA to preserve their phenotype (CD25^HI^, FOXP3^+^) and the levels of FOXP3 (Figure 8), suggesting that the use of *in vitro* generated allospecific iTregs in adoptive therapy will require the use of RAPA to ensure their stability and efficacy *in vivo* (45). In addition, immunomodulatory agents for *in vivo* iTreg generation can be considered such as hormone erythropoietin, as its application to patients with chronic kidney disease resulted in an increase of Tregs after 6 months of treatment. In this context, *in vitro* assays confirmed that erythropoietin has an effect on APCs inducing the production of TGF-β1, an important cytokine for iTreg generation (46).

To date, there are a few published protocols on *in vitro* generation of human Tregs, most of them polyclonal and in short-term cultures (1–2 weeks) and some of them with no data on the methylation status of the FOXP3 gene (47–49). Other reports on polyclonal conversion showed that FOXP3 remains methylated or that Treg restimulation causes the loss of its expression (23, 24, 50). Among the studies describing long-term generation of allospecific iTregs, Tu et al. reported the use of CD40L-stimulated B cells (NIH 3T3 cells transfected with the CD40 ligand; tCD40L NIH/3T3) as antigen-presenting cells. Although they obtained up to 8.3 × 10^6^ per million “naive” at 21 days of culture with 92% purity, iTregs required to be continuously expanded by weekly addition of B-CD40L cells (37), and the presence of contaminating cells in the co-cultures affected the purity of the generated iTregs. Finally, none of the reports evaluated the stability of the iTregs generated in inflammatory conditions, as presumably would occur in a transplant scenario.

Stable expression of FOXP3 in Tregs depends on the demethylation status in a conserved region rich in CpG within the FOXP3 locus, called TSDR, which is not required for the initiation of FOXP3 expression but for its maintenance (51). When we analyzed the methylation status of TSDR FOXP3, we found a partial demethylation (≈40%) of iTregs recently isolated from co-cultures with Mo-DCs; however, this demethylation was diminished when the cells were expanded for 4 or 6 weeks with anti-CD3/anti-CD28-coated beads, although no methylation was not complete as in naïve T cells (Figure 9). These results might suggest that the allo-iTregs generated might not be stable; however, the increasing expression of FOXP3 during the course of the expansion and the maintenance of their phenotype and function in the presence of inflammatory cytokines are in discrepancy with their methylation status. One study reported no increase in FOXP3 expression and suppressive function in iTregs when they were treated with the inhibitor of DNA methyltransferase Decitabine, suggesting that FOXP3 methylation status does not control the induction of Tregs (49). It was reported that epigenetic changes required for the stability of iTregs are different from those in tTregs. For example, FOXP1 transcription factor is required for the optimal expression of FOXP3 in iTregs but is independent of the cell division and methylation status of the TSDR FOXP3 region. Moreover, the absence of FOXP1 does not affect the methylation status of FOXP3, but rather the chromatin modification of the FOXP3 locus (52). In addition, in TGF-β1-induced iTregs, there is an increase in the trimethylation of histone H3K4, even in the absence of hypomethylation (53). Also, it has been reported that some transcription factors such as C/EBP require methylated TSDR to ensure stable induction of FOXP3 in iTregs and, in this way, protect FOXP3 expression from inhibitory cytokines at the early stages of its development (54).

Some reports indicate that demethylation in iTregs is a process that is gradually acquired during the course of their differentiation or that can be acquired *in vivo* after being transferred into an organism. Chen et al. reported that *in vitro*-generated iTregs acquire TSDR demethylation *in vivo* only after treatment of mice with IL-2 (55). It is interesting to point out that some clones acquired total demethylation TSDR, so the refinement in their characterization could improve the isolation of stable iTregs with an established TSDR demethylation pattern over the course of the culture. In this context, recent reports have shown that is possible to increase the demethylation status of the TSDR region by adding vitamin C to iTreg cultures (56, 57), which was reported to be an activator of TET enzymes, which, in turn, control the stability of FOXP3 (47).

In conclusion, the methodology described here allows a sufficient number of allospecific iTregs with stable phenotype and antigen-specific suppressor function in the presence of pro-inflammatory cytokines, with therapeutic potential in a transplant setting.

## Data Availability Statement

The raw data supporting the conclusions of this article will be made available by the authors, without undue reservation, to any qualified researcher.

## Ethics Statement

The studies involving human participants were reviewed and approved by The Ethics Research Commitee at the Instituto Nacional de Ciencias Médicas y Nutrición Salvador Zubirán (Reference number 1831) and the CEISHUM Commitee at the Instituto de Investigaciones Biomédicas (UNAM). The patients/participants provided their written informed consent to participate in this study.

## Author Contributions

EA-S performed experiments, analyzed data, and wrote the manuscript. AC-H performed experiments and revised the manuscript. SA-C performed experiments. JA-G designed the study and reviewed the manuscript. GS supervised the research, analyzed the data, wrote the manuscript, and obtained funding.

### Conflict of Interest

The authors declare this manuscript have conflicts of interest to disclose as described by *Frontiers of Immunology*. EA-S, AC-H, JA-G, and GS have a provisional application filed on allospecific iTregs (MX/a/2019/012911). The remaining author declares that the research was conducted in the absence of any commercial or financial relationships that could be construed as a potential conflict of interest.
