# Supplementary figures and images for: Large-Scale Generation of Human Allospecific Induced Tregs With Functional Stability for Use in Immunotherapy in Transplantation
Source: Front Immunol. 2020 Apr 2;11:375. doi: 10.3389/fimmu.2020.00375 (PMC7142244; doi:10.3389/fimmu.2020.00375)

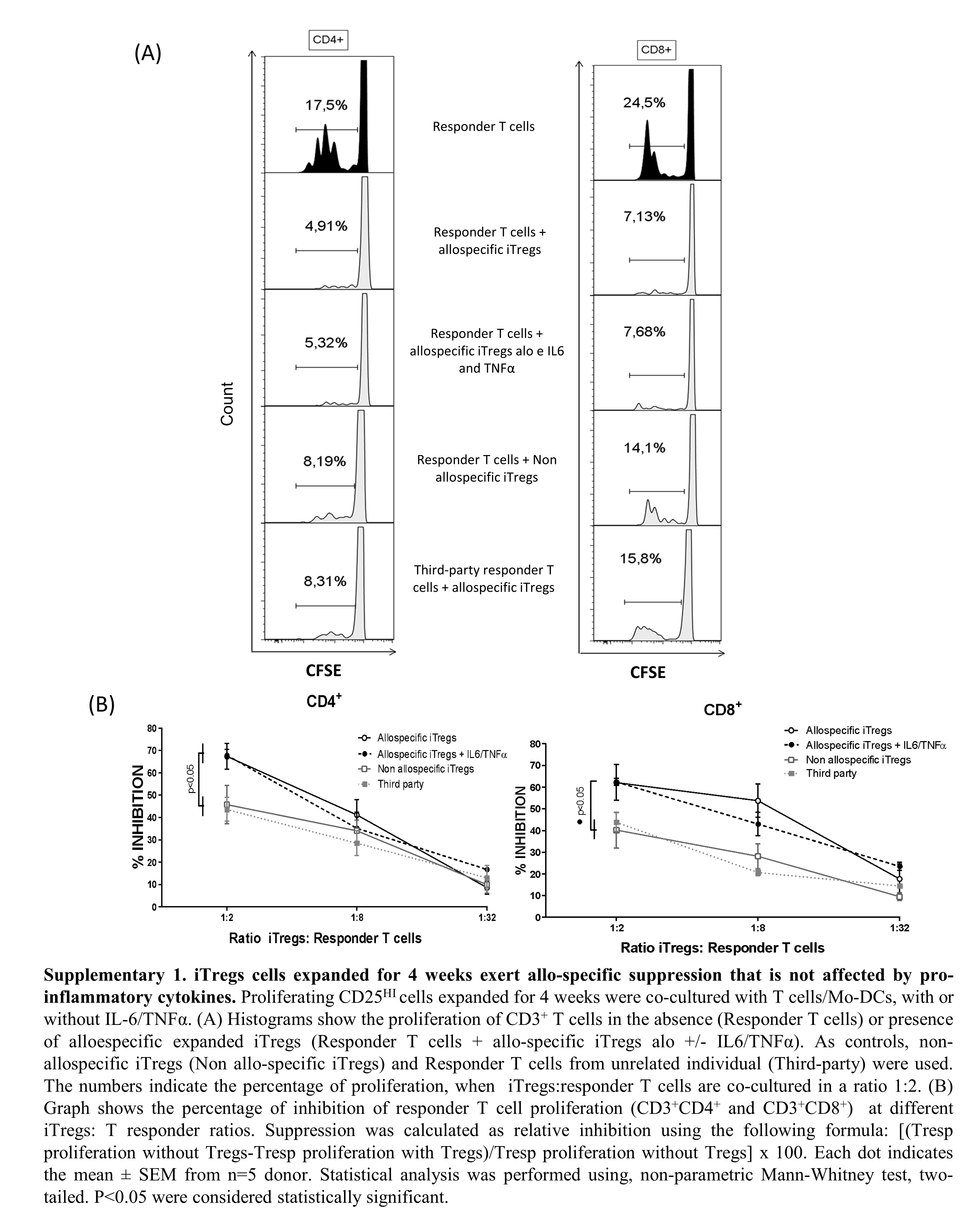

Supplement: Supplementary file 1 [file Image_1.tiff]

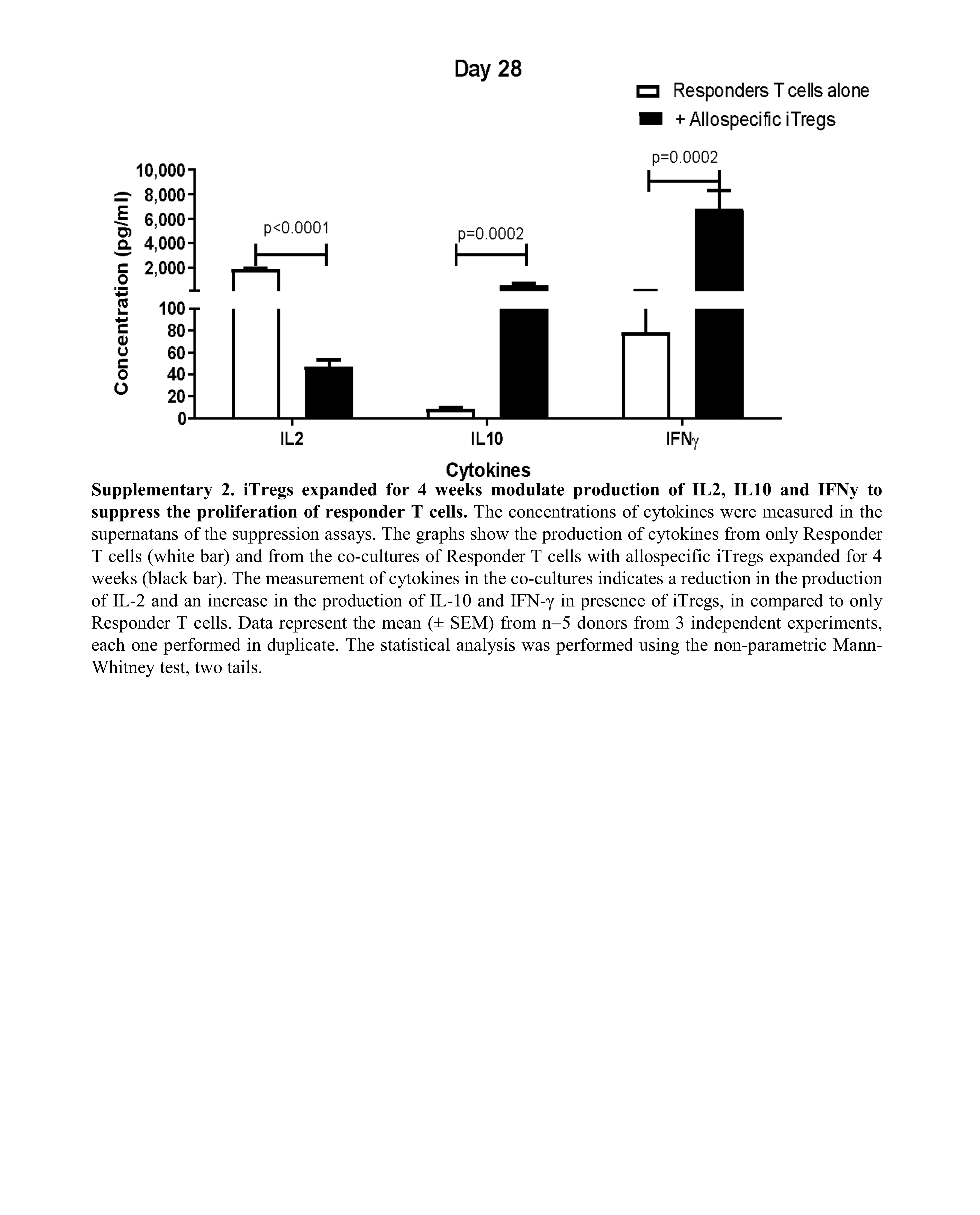

Supplement: Supplementary file 2 [file Image_2.TIF]
